# Transcriptomics insights into the functional role of tick *Ixodes ricinus* proteins metalloprotease and antigen p23

**DOI:** 10.1371/journal.pone.0336570

**Published:** 2025-11-14

**Authors:** Rita Vaz-Rodrigues, Vincent C. Duru, Ard M. Nijhof, José de la Fuente

**Affiliations:** 1 SaBio (Health and Biotechnology), Instituto de Investigación en Recursos Cinegéticos (IREC, CSIC-UCLM-JCCM), Ciudad Real, Spain; 2 Institute of Parasitology and Tropical Veterinary Medicine, Freie Universität Berlin, Berlin, Germany; 3 Veterinary Center for Resistance Research, Freie Universität Berlin, Berlin, Germany; 4 Department of Veterinary Pathobiology, Center for Veterinary Health Sciences, Oklahoma State University, Stillwater, Oklahoma, United States of America; University of Missouri College of Veterinary Medicine, UNITED STATES OF AMERICA

## Abstract

*Ixodes ricinus* ticks (Acari: Ixodidae) are hematophagous ectoparasites and a major European vector of zoonotic diseases affecting global health. Tick salivary and midgut proteins antigen p23 (A0A0K8RKR7) and metalloprotease (A0A0K8RCY8) were previously implicated in the pathophysiology of the tick-borne allergy alpha-Gal syndrome (AGS). This study aimed to functionally characterize these two biomolecules, focusing on their role in *I. ricinus* tick feeding and reproduction through gene knockdown by RNA interference and midgut transcriptomic analysis. Validation of RNA-seq data was conducted using RT-qPCR analysis on tick midgut and salivary gland tissues. Silencing the expression of p23 and metalloprotease did not result in any significant differences in tick engorgement and egg batch weights compared to the control group. Gene set enrichment analysis following antigen p23 gene knockdown identified significantly upregulated pathways associated with protein production and suppressed routes mostly correlated with ion transport, lipid metabolism, catalytic activity, protein modification, and G-protein activity. Partial knockdown of the metalloprotease led to the upregulation of several biological and functional pathways associated with RNA splicing and significantly suppressed routes connected with detoxification, protein modification, catalytic activity and molecule binding. Antigen p23 appears to play a functional role in tick midgut cell homeostasis, primarily by participating in regulatory and signaling processes essential for cell viability. Metalloprotease is potentially involved in regulating midgut response to oxidative stress, thereby reducing tissue damage and promoting regular cell proliferation, growth and behavior. These results provide insights into tick physiology and bases for further research on tick-host interactions and AGS pathogenesis.

## Introduction

Ticks are obligate hematophagous ectoparasites responsible for the transmission of infectious agents, affecting human and animal health worldwide [1,2]. Ticks are the second most important arthropod vectors after mosquitoes, harboring and transmitting a broader range of medically relevant pathogens, including bacteria, viruses, protozoa, and helminths [3,4]. Climate change is expected to alter the tick-host dynamic, leading to a rise in tick-borne disease cases in previously unaffected regions, presenting new public health challenges in the future [5,6]. The castor bean tick, *Ixodes ricinus,* is the main European vector of pathogens causing major zoonotic diseases, tick-borne encephalitis (TBE) caused by a Flavivirus and Lyme borreliosis (LB) caused by genospecies of *Borrelia burgdorferi* sensu lato (s.l.) [7,8]. Occasionally, tick-host interactions can lead to the development of emergent tick-borne allergies like the alpha-Gal syndrome (AGS), with bites from *I. ricinus* in Europe being primarily associated with the onset of this allergy [9,10].

During the blood-feeding process, ticks can potentially transmit and acquire pathogens, with tick saliva and the midgut acting as central components of the host-vector-pathogen interface [11,12]. While essential for blood meal processing and nutrient storage, the tick midgut is also a key organ involved in the transmission of pathogens like the causative agent of LB [13]. These processes are mainly driven by midgut proteins that take part in blood digestion and enable pathogen transmission, making them promising candidates for novel drug and anti-tick vaccine development [11,14]. Although AGS pathogenesis is more directly associated with tick salivary proteins [9], alpha-Gal antibodies were found to cross-react with the unfed midgut of *Ixodes scapularis* females, suggesting the presence of molecules bound to alpha-Gal in this tissue [15]. Metalloprotease (UniProt ID: A0A0K8RCY8) and antigen p23 (UniProt ID: A0A0K8RKR7) are both salivary gland and midgut proteins with anticoagulant activity, known to promote optimal tick feeding [16–18] and potentially implicated in AGS [19,20]. However, additional physiological role of these proteins in ticks remains unknown.

RNA interference (RNAi), a conserved post-transcriptional gene-silencing mechanism, has proven invaluable for studying tick protein functions at tick-host interface, especially those involved in feeding or pathogen transmission [21–23]. Animal models have been extensively used in biomedical and translational research to better understand the pathogenesis of tick-borne diseases [24,25]. Although significant, the use of live animal hosts in traditional tick research faces challenges, including ethical concerns and increased variability in host responses [26]. As research on tick-borne diseases advances, alternative methods like artificial membrane feeding systems are becoming increasingly crucial in overcoming the limitations of traditional approaches. This method offers a controlled alternative that reduces ethical dilemmas while enabling precise monitoring of tick attachment, feeding, and pathogen acquisition [26,27].

Despite recent advances in tick physiology research, current findings highlight a knowledge gap regarding the functional roles of key tick midgut proteins. Therefore, the aim of this study was to functionally characterize biomolecules antigen p23 (UniProt ID: A0A0K8RKR7) and metalloprotease (UniProt ID: A0A0K8RCY8) based on their role in *I. ricinus* tick feeding and reproduction, determined after gene knockdown by RNAi and further midgut transcriptomics (mialome) analysis. Validation of RNA-seq data was further conducted using RT-qPCR analysis on tick midgut and salivary gland tissues.

## Materials and methods

### Ticks

All *I. ricinus* ticks used in the study originated from a laboratory colony maintained at the Institute for Parasitology and Tropical Veterinary Medicine, Freie Universität Berlin. Ethical approval for the maintenance of the *I. ricinus* colony is provided by the regional authorities for animal experimentation “Landesamt für Gesundheit und Soziales” (LAGeSo) of Berlin, under registration number E0144/22.

### Total RNA extraction and synthesis of tick cDNA for dsRNA preparations

The midguts of five *I. ricinus* females, partially fed *in vitro* for three days with bovine blood [28], were dissected and immersed in 500 µl of TRI Reagent (Sigma-Aldrich, Taufkirchen, Germany). Total RNA was extracted and subsequently purified using the Direct-Zol RNA Miniprep Plus Kit (Zymo Research, Freiburg im Breisgau, Germany), in accordance with manufacturer’s guidelines. Total RNA concentration and purity was determined using a Synergy HT Spectrophotometer (Bio-Tek Instruments, Bad Friedrichshall, Germany) and samples were stored at −80 °C prior to use. Complementary DNA (cDNA) was obtained using a ProtoScript II First Strand cDNA synthesis kit (New England Biolabs, Ipswich, USA), following manufacturer’s instructions. Afterwards, the cDNAs were used as template for dsRNA synthesis.

### Generation of dsRNA

Oligonucleotide forward (F) and reverse (R) primers (Sigma-Aldrich) containing T7 promoter sequences (5’-TAATACGACTCACTATAGG-3’) at the 5’-end to support *in vitro* transcription and synthesis of dsRNA were used to PCR-amplify partial cDNA fragments of genes encoding *I. ricinus* antigen *p23* (386 bp; F: T7-ACTACAAGTGAACGTGTCTCGG; R: T7-ACACGGTCAGAACCTTGTCC) and *metalloprotease* (481 bp; F: T7-AGCTGAAGACGCTGAACGTG; R: T7-CTCGTCCGCTGAGTTTTTGT). Gene-specific primers were designed using NetPrimer (Premier Biosoft International) and primer–BLAST online tools [29]. An unrelated sequence coding for green fluorescent protein (GFP; 415 bp; F: T7-GGCCACAAGTTCAGCGTGTC; R: T7-GCTTGATGCCGTTCTTCTGC) was used as a negative control [30,31]. PCR amplicons were purified using the DNA Clean and Concentrator kit (Zymo Research), according to manufacturer’s recommendations and sequenced using the Sanger method at LGC Genomics GmbH (Berlin, Germany). Low-quality sequences were trimmed from both ends using Chromas software (version 2.6.6) and subsequently used to confirm correct amplification. BLAST analysis for the p23 amplicon (NCBI GenBank accession no. PV761653) revealed 97% nucleotide sequence identity with *I. scapularis* protein antigen p23 sequence deposited in NCBI GenBank (accession no. HQ605984). BLAST analysis for the metalloprotease amplicon (NCBI GenBank accession no. PV761654) showed 95% nucleotide sequence identity with a *I. ricinus* mRNA sequence coding for the Metis1 protein deposited in NCBI GenBank (accession no. AM747806). Therefore, these PCR products were used as templates to produce dsRNA using the T7 Ribomax Express RNAi system (Promega, Walldorf, Germany), following manufacturer’s guidelines. The dsRNA was quantified by spectrophotometry and stored at −80 °C until further use.

### RNA interference by tick injection with dsRNA

Unfed *I. ricinus* adult female ticks (n = 240) were randomly assigned into three groups (n = 80 per group) and injected with approximately 0.5 µL of dsRNA (1 x 10^12^ molecules/µL) coding for one of the selected targets or GFP dsRNA as a negative control. The injections were performed in the lower right quadrant of the ventral surface of the exoskeleton using a 10 µL syringe with a 33-gauge needle (Hamilton, Bonaduz, Switzerland) mounted on a micromanipulator. Following dsRNA injection, ticks were distributed into different feeding units (four units per group), each containing 20 *I. ricinus* females and 10 males, for artificial feeding on bovine blood. Feeding units were made and set up as previously described [28]. The blood meal consisted of 5 mL of defibrinated bovine blood per feeding unit supplemented with adenosine triphosphate (ATP, 51 mg/mL), glucose (3 mg/mL), B vitamin (10 µl/mL) and gentamycin (1 µg/mL) [32]. Ticks were maintained at an internal ambient temperature of 27°C with 70% relative humidity, 1% CO_2_ and 50% ventilation. The feeding units were placed on a hotplate set to 42°C until the ticks reached full engorgement (12.17 ± 1.98 days). The blood meals were changed twice daily, and each feeding unit was cleaned before re-immersion into fresh blood meals with 0.9% NaCl solution. Data on the number of dead and feeding females was recorded twice daily. The experiment lasted 15 days. Engorged females that detached were weighed and stored individually. Engorgement and egg batch weights were also recorded. Values of antigen p23 and metalloprotease tick groups (tick engorgement and eggs weight) were compared with the GFP control group by one-way ANOVA test with post-hoc Tukey HSD test using the Real Statistics extension from Microsoft Excel (version 2310) and graphically represented with Graph-Pad Prism (version 8.0.1).

### Corroboration of gene knockdown by reverse transcriptase quantitative PCR (RT-qPCR)

After seven days of artificial feeding, the midguts and salivary glands were collected from female ticks in each group (n = nine/group), all of which had comparable engorgement sizes. The samples were analyzed in three biological replicates per group, with each replicate consisting of three ticks. Total RNA extraction was performed using TRI Reagent (Sigma-Aldrich, Taufkirchen, Germany), following manufacturer’s instructions. Genomic DNA was removed from RNA samples using the rDNase Set (Macherey-Nagel, Dueren, Germany) and repurified with the NucleoSpin® RNA Clean-up XS (#740903, Macherey-Nagel, Dueren, Germany), following manufacturers’ recommendations. The concentration (ng/µL) and purity of samples were assessed using a Nanodrop One spectrophotometer (Thermo Scientific, Waltham, USA) by measuring nucleic acid absorbance at 260 nm (OD260) and the 260/280 nm ratio. Concentrations were standardized at 50 ng/µL and the reverse transcription of total mRNA into cDNA was performed using the ProtoScript II First Strand cDNA synthesis kit (New England Biolabs, Ipswich, USA). Gene silencing levels of antigen p23 and metalloprotease after RNAi were assessed by qPCR on cDNA samples, using gene-specific oligonucleotide primers designed to amplify a distinct mRNA fragment from those targeted by the dsRNAs (antigen p23, 125 bp, F: TAAGCCTTCTCACGCTGTCT, R: AATGGGCTCCGACGTGATCA; metalloprotease, 158 bp, F: CATCAGCGAGCAACTATTGC, R: TCCCTTTCCGCAAGGTGTAC). Amplification of synthetized cDNA was performed using the Luna Universal qPCR Master Mix (New England Biolabs, Ipswich, USA) and the CFX96 real-time PCR detection system (Bio-Rad, Hercules, USA). qPCR conditions comprised an initial denaturation step at 95 °C for 1 min, amplification by 40 cycles of 95 °C for 20 s and different annealing temperatures for 1 min (antigen p23: 58 °C, metalloprotease: 54 °C), followed by a dissociation curve analysis. The tick elongation factor 1-alpha (elf1a, 106 bp, F: CAAGATTGGTGGTATCGGCA, R: GACCTCAGTGGTGATGTTGGC) was the housekeeping gene used to normalize the expression of the targets analyzed, applying the Delta-Delta-Ct (ΔΔCt) method [33]. Cycle threshold (Ct) values for normalized antigen p23 and metalloprotease dsRNAs samples were compared with those of the GFP control samples using the Kruskal–Wallis test followed by Dunn’s multiple comparisons post-hoc test in Graph-Pad Prism (version 8.0.1) to determine significant levels of gene knockdown.

### Transcriptome: RNA extraction, library construction, and sequencing

At day seven of artificial feeding, total RNA from three groups (antigen p23, metalloprotease and GFP control) was extracted employing 500µl of TRI Reagent (Sigma-Aldrich, Taufkirchen, Germany), following manufacturer’s instructions. Samples included the midguts of nine *I. ricinus* female ticks per group divided into three biological replicates, each containing three ticks per pool. Genomic DNA was removed from RNA samples using the rDNase Set (Macherey-Nagel, Dueren, Germany) and repurified with the NucleoSpin® RNA Clean-up XS (#740903, Macherey-Nagel, Dueren, Germany), following manufacturer’s recommendations. RNA concentration and purity were assessed using a Nanodrop One spectrophotometer (Thermo Scientific, Waltham, USA). RNA integrity was evaluated with the RNA 6000 Nano kit on a Bioanalyzer 2100 system (Agilent Technologies, CA, USA).

Library preparation and paired-ended 150 bp (PE150) sequencing were performed at Novogene GmbH (Munich, Germany). Sequencing libraries were generated using the Novogene NGS RNA Library Prep Set (PT042; Novogene, Beijing, China), following manufacturer’s instructions. Index codes were incorporated to associate sequences with their corresponding samples. Briefly, mRNA was purified from total RNA using poly-T oligo-attached magnetic beads (poly-A-tailed enrichment). After random mRNA fragmentation, the first strand of cDNA was synthesized using random hexamer primers. Second strand cDNA was then performed employing an Illumina buffer containing dNTPs, DNA Polymerase I, and RNase H. The remaining overhangs were converted to blunt ends by exonuclease and polymerase activities. Following adenylation of the 3’ ends of cDNA fragments (A-tailing), a hairpin-loop shape adaptor was ligated in preparation for hybridization. The uracil-specific excision reagent (USER) enzyme was used to preferentially select cDNA fragments of 150 ~ 200 bp in length, following manufacturer’s recommendations. Library quality assessment was performed using the Qubit® 2.0 Fluorometer (Thermo Scientific, Waltham, USA) for concentration measurement, qPCR for quantification, and the Bioanalyzer 2100 system (Agilent Technologies, CA, USA) for size distribution analysis after purification. Quantified libraries were pooled and sequenced on the Illumina NovaSeq X Plus system, generating between 82.3 million and 244.2 million total raw reads per library in the RNA sequencing dataset. Transcriptomics data was deposited in the NCBI Gene Expression Omnibus (GEO) database, under the GEO series accession no. GSE291137.

### Bioinformatics, quantification and differential gene expression

Raw reads on FASTQ format were processed using in-house Perl scripts to generate clean reads by removing adapter sequences, poly-N reads, and low-quality reads. The dataset yielded high-quality data, consisting of approximately 81.0–240.3 million clean reads for each library, which translates to 97.7–98.5% of the raw reads being clean reads. Additionally, Q20 scores ranged from 97.9% to 98.3%, while Q30 scores varied between 94.0% and 95.0%. Mapping was performed using the *I. persulcatus* genome (assembly BIME_Iper_1.3) serving as reference and Bowtie 2 (v2.5.3) as the alignment tool [34,35]. Direct mapping of the assembled *I. ricinus* reads against the *I. persulcatus* genome yielded a mapping rate ranging from 45.3% to 47.6%. Furthermore, exon-mapped reads accounted for 48.6% to 53.7% of the mapped regions, indicating a well-annotated reference genome. The final BAM files were quantified with the *featureCounts* function available in Bioconductor R package *Rsubread* [36] using BIME_Iper_1.3 annotations. Raw count units were normalized using the FPKM method, which accounts for variations in gene length and sequencing depth, enabling the estimation of gene expression levels.

Differential expression analysis of sequence data (DESeq) was conducted using the *DESeq2* package (v1.46) [37] from Bioconductor (v3.20) in R software (v4.4.2) [38]. The normalized count data are fitted to a negative binomial generalized linear model to analyze differences between conditions (antigen p23 vs. GFP control, and metalloprotease vs. GFP control). The model identifies differentially expressed genes (DEGs), with the significance threshold set at a Benjamini-Hochberg (BH) adjusted p-value cutoff of ≤ 0.05 and a Log2 Fold Change (Log_2_FC) ≥ |0|. Additionally, the volcano plots, heatmap clustering, and Venn diagram highlighting the DEGs per treatment group were created in R [38] employing the *ggplot* [39], *heatmap* and *venn.diagram* functions, respectively.

### Functional annotation and enrichment analysis

The functional prolife of significant transcripts was obtained using the gene ontology (GO) biological process (BP), molecular function (MF) and cellular component (CC) databases from InterPro2GO annotations [40] available through the Eukaryotic Pathogen, Vector, and Host Informatics Resources (VEuPathDB) [41]. Gene set enrichment analysis (GSEA) was conducted using the GO database with the *gseGO* function from the *clusterProfiler* package [42,43]. The analysis employed 1,000 permutations and a BH adjusted p-value cutoff of 0.05 to identify significant biological pathways. GSEA analysis for the Kyoto Encyclopedia of Genes and Genomes (KEGG) pathways was carried out with the *gseKEGG* function from the *clusterProfiler* package [42,43]. The analysis utilized 1,000 permutations and a BH adjusted p-value cutoff of 0.05 to determine significant biochemical pathways. The software Graph-Pad Prism (version 8.0.1) was applied to visually represent the results of the functional enrichment analysis.

### Corroboration of transcriptomics results by RT-qPCR

Eight target DEGs (Tables 1 and 2) were selected based on their predicted biological function. Their expression levels were measured in female *I. ricinus* tick salivary and midgut samples (three biological replicates per group, each consisting of pooled tissues from three ticks; n = 9 per group) via the amplification of synthesized cDNA using the CFX96 real-time PCR detection system (Bio-Rad, Hercules, USA). Gene-specific primers were designed using the primer–BLAST [29] online tool and are listed in S1 and S2 Tables in S1 File. The reverse transcription of total mRNA (5 µl/sample) into cDNA was performed using the iScript cDNA Synthesis Kit (Bio-Rad, Hercules, USA), following the guidelines of the manufacturer. The cDNA sample concentrations were standardized to 200 ng/μL. Quantitative PCR conditions comprised an initial denaturation step at 95 °C for 1 min, amplification by 40 cycles of 95 °C for 20 sec and annealing between 58–62 °C for 1 min, followed by a dissociation curve analysis. For a total volume of 20 µL, the PCR mixture contained 1.5 μL (300 ng) of sample cDNA, 10 μL of SYBR Green Master Mix (Bio-Rad), 1 μL (10 μM) of forward and reverse primers (Sigma-Aldrich) each and 6.5 μL of nuclease-free water. Each PCR reaction had 2 technical replicates/sample and 2 negative controls. The Delta-Delta Ct method [33] was applied to calibrate, normalize, and determine the relative expression levels of the gene knockdown groups (antigen p23 or metalloprotease) against the reference GFP control samples. The housekeeping gene used was the tick elongation factor 1-alpha (elf1a, 141 bp, F: AACGGCTACACGCCTGTTCT, R: GATGGCAGCATCTCCGGACT). Statistical comparisons between groups were performed with the non-parametric Mann-Whitney U test using the Real Statistics extension from Microsoft Excel (version 2310). Data expressed as fold change was depicted utilizing GraphPad Prism (version 8.0.1).

**Table 1:** Selected targets for transcriptomics data validation via RT-qPCR following antigen p23 gene silencing.

Target	Gene ID	LogFC	BH p-value	Function prediction
Ribosomal protein L24 (RPL-24)	HPB47_017850	0.72	0.047	Translation and RNA binding within the ribosome
Aurora kinase-like (Aur)	HPB47_014629	−3.15	0.002	Protein tyrosine activity (phosphorylation) and ATP binding
Allatostatin type A receptor (AstA)	HPB47_005012	−5.00	0.044	G-protein coupled receptor signaling pathway in cell membrane
Mitochondrial phosphatidate cytidylyltransferase (CDS)	HPB47_023844	−3.20	0.049	Lipid metabolic process (cardiolipin biosynthesis) and nucleotidyltransferase activity

BH – Benjamini-Hochberg; FC – fold change.

**Table 2:** Selected targets for transcriptomics data validation via RT-qPCR following metalloprotease gene knockdown.

Target	Gene ID	LogFC	BH p-value	Function prediction
Ribonuclease T2 like (RNase T2)	HPB47_001654	3.08	0.009	Ribonuclease activity and RNA binding
Mediator complex subunit 13 (MED13)	HPB47_027399	−0.79	0.038	Transcription coregulator activity (regulation by RNA polymerase II)
Guanylate cyclase (GC)	HPB47_008153	−2.10	0.005	Protein tyrosine activity (phosphorylation) and ATP binding
Cytochrome P450 (CYP450)	HPB47_019282	−2.26	0.005	Oxidoreductase activity (monooxygenase) with heme and iron binding

BH – Benjamini-Hochberg; FC – fold change.

## Results

### Phenotypic changes with proteins p23 and metalloprotease gene knockdown

**Fig 1:**
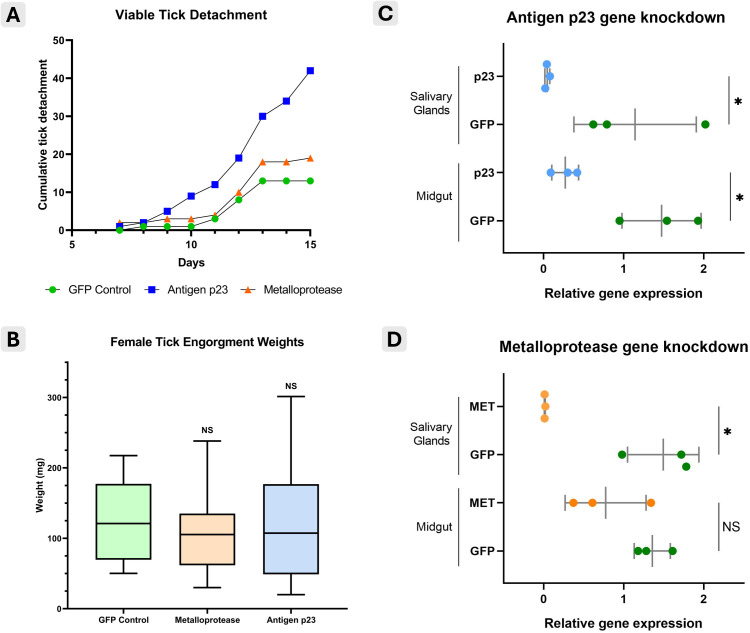
Phenotypic changes with proteins antigen p23 and metalloprotease gene knockdown. **(A)** Cumulative number of viable ticks detached over the 15-day experiment reported daily for each group. **(B)** Boxplot of female tick engorgement weights once detached and reported per group. **(C)** Corroboration of gene knockdown in group injected with dsRNA coding for antigen p23 using salivary gland and midgut tissues. **(D)** Confirmation of gene knockdown in group injected with dsRNA targeting metalloprotease in salivary glands and midgut. * p < 0.05, ** p < 0.01, *** p < 0.001, NS: not significant.

Initial mortality following tick dsRNA injections and allocation to a feeding unit was low, not exceeding 3.33%, with two to three dead ticks per group. Feeding behavior analysis showed that tick attachment peaked on day 4. In the antigen p23 and metalloprotease groups, 68.8% (55/80) of the female ticks attached, compared to 62.5% (50/80) in the GFP control group. Of the attached ticks, 76% (42/55) in the antigen p23 group, 33% (18/55) in the metalloprotease group, and 24% (12/50) in the GFP control group successfully engorged and detached from the host. Cumulative tick detachment per group throughout the experimental period is shown in Fig 1A.

Silencing the expression of antigen p23 and metalloprotease did not result in any significant differences in tick engorgement weights compared to the GFP control group (Fig 1B), although the average weights were slightly lower. Oviposition was scarce, with only 21.4% (9/42) of ticks in the antigen p23 group, 16.7% (3/18) in the metalloprotease group, and 25% (3/12) in the GFP control group successfully laying eggs. The average egg batch weights showed no significant differences between groups: antigen p23 (20.3 mg), metalloprotease (10 mg), and GFP control (21.7 mg). Antigen p23 gene knockdown was successful in both tick salivary gland (GFP mean Ct = 22.91 ± 1.00 vs. p23 mean Ct = 28.05 ± 1.41) and midgut (GFP mean Ct = 21.35 ± 0.91 vs. p23 mean Ct = 27.14 ± 1.31) tissues (Fig 1C). Interestingly, metalloprotease was fully silenced only in the salivary gland tissue (GFP mean Ct = 22.34 ± 1.66 vs. MET mean Ct = 27.74 ± 0.52), but not in the midgut (GFP mean Ct = 22.95 ± 0.56 vs. MET mean Ct = 25.22 ± 1.16; Fig 1D).

### Differential expression analysis and descriptive functional profile

**Fig 2:**
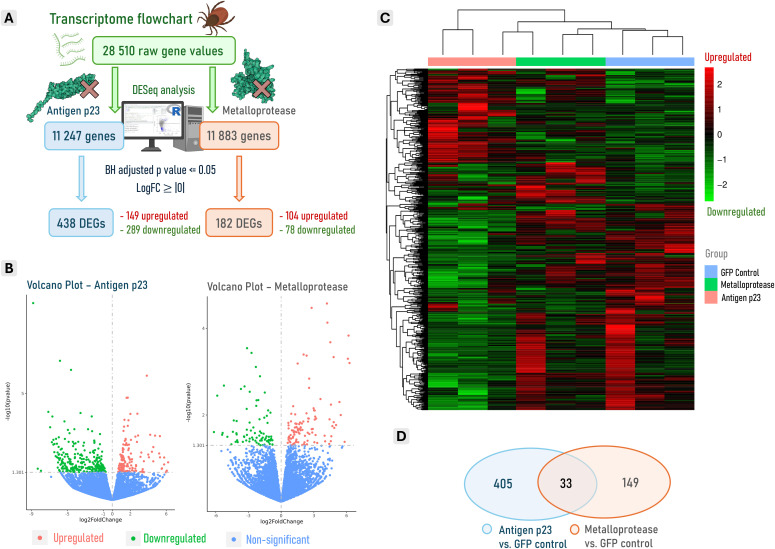
Transcriptome pipeline and visual representation of gene expression from *Ixodes ricinus* tick midgut underlying the effects of silencing the expression of antigen p23 and metalloprotease by RNAi. **(A)** Transcriptome flowchart that leads to the acquisition of differentially expressed genes (DESeq) after gene knockdown. **(B)** Volcano plots illustrating the upregulated and downregulated DEGs following the silencing of antigen p23 and metalloprotease in tick midguts. **(C)** Heatmap clustering by expression patterns observed in the experimental groups (antigen p23, metalloprotease and GFP control). **(D)** Veen diagram showcasing DEGs similarities and differences between antigen p23 and metalloprotease groups.

After subjecting 28,510 raw gene count units to DESeq analysis and applying various filters (Fig 2A), a total of 438 differentially expressed genes (DEGs) were found in the antigen p23-silenced group compared to the GFP control group, including 149 upregulated and 289 downregulated genes (Fig 2B, S1 Data). Meanwhile, silencing metalloprotease in tick midguts led to the identification of 182 DEGs, comprising 104 upregulated and 78 downregulated genes (Fig 2B, S2 Data). Gene clustering by group revealed greater expression differences in the antigen p23 group, while the metalloprotease and GFP control groups exhibited closer expression patterns (Fig 2C), likely due to the absence of effective metalloprotease gene knockout in the tick midgut. Additionally, the overlapping regions of the Venn diagram indicated that silencing both antigen p23 and metalloprotease led to the co-expression of 33 DEGs (Fig 2D). Of these, 84.8% (28/33) exhibited similar expression patterns, including 12 upregulated and 16 downregulated genes.

Upregulated DEGs after antigen p23 silencing in tick midgut were mostly associated with the biological process (BP) of proteolysis (n = 7). Among these, the molecular function (MF) was predominantly represented by serine-type peptidases (n = 3; HPB47_004434, HPB47_005579, HPB47_014190) and metallopeptidases (n = 2; HPB47_009280, HPB47_024456). Silencing antigen p23 primarily impacts the downregulation of transmembrane transport processes (n = 18), including five genes associated with ion transmembrane transport (S3 Table in S1 File). Another key biological process affected was the suppression of G protein-coupled receptor signaling pathway (n = 9). Additionally, protein binding (n = 34) appears to be the most affected molecular function resulting from the knockdown of antigen p23 gene. Other gene ontology (GO) processes of interest related to antigen p23 gene knockdown can be found in S3 Table in S1 File.

The partial knockdown of metalloprotease in the midgut led to the upregulation of DEGs associated primarily with the transmembrane transport (n = 8) BP. The GO MFs of upregulated genes were mostly linked to sulfotransferase (n = 6) and oxidoreductase (n = 5) activities (S4 Table in S1 File). On the other hand, downregulated genes were not only connected to proteolysis (n = 4) but also with ion transmembrane transport (n = 3) as part of the BPs, whereas MF processes revealed a suppression in protein binding (n = 7). Additional GO processes of interest related to metalloprotease gene knockdown can be found in S4 Table in S1 File. Genes co-expressed as a result of proteins p23 and metalloprotease gene knockdown were predominantly associated with GO terms such as ion transmembrane transport, proteolysis, and protein binding.

### Enrichment analysis after silencing antigen p23 in tick midgut

**Fig 3:**
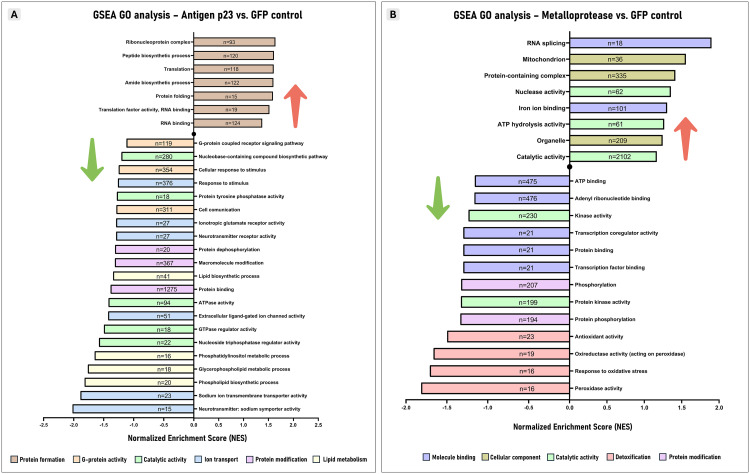
Tick gut transcriptome enrichment analysis following the ablation of endogenous antigen p23 (A) and metalloprotease (B) by RNAi. **(A)** Bar plot of gene set enrichment analysis (GSEA) using the gene ontology (GO) biological process (BP), molecular function (MF) and cellular component (CC) databases, following antigen p23 gene knockdown. **(B)** Bar plot of GSEA method using the GO BP, MF and CC databases, following metalloprotease gene knockdown. These plots highlight the most relevant and specific GO terms for each enriched gene set, separated by upregulated (positive NES values) and downregulated (negative NES values) pathways when comparing the silenced groups to the GFP control group. All enriched pathways can be found in S1 and S2 Datas.

The GSEA method for GO BP, MF and CC terms yielded a total of 96 significantly enriched pathways, including 9 upregulated and 87 downregulated routes (S1 Data). This GSEA analysis following antigen p23 gene knockdown identified significantly upregulated pathways associated with protein production (Fig 3A). These routes included the activation of GO terms such as protein folding, translation, and RNA binding within the ribonucleoprotein complex. Significantly suppressed biological and functional pathways were categorized into one of the following five groups: ion transport, lipid metabolism, catalytic activity, protein modification, and G-protein activity (Fig 3A). Ion transmembrane transport accounted for the pathway with the lowest normalized enrichment score (NES, −2.02), which corresponded to the suppression of neurotransmitter sodium-symporters activity. The suppression of lipidic biosynthetic processes (NES = −1.34) was mainly related to a reduction in the production of glycerophospholipids (NES = −1.76), particularly phosphatidylinositol (PI, NES = −1,82). Catalytic activity was driven by the suppression of protein tyrosine phosphatase (PTP) activity (NES = −1.28), which catalyzes dephosphorylation (NES = −1.31), a key protein modification mechanism. The downregulation observed in cell communication (NES = −1.29) and response to stimuli (NES = −1.25) seems to be associated with a reduction in G-protein-coupled receptor (GPCR) signaling pathway activity (NES = −1.12). Furthermore, the GSEA KEGG analysis identified four significantly enriched biochemical pathways (S1 Data), with three activated pathways including phagosome (NES = 1.46), nucleotide excision repair (NER, NES = 1.38) and nucleocytoplasmic transport (NCT, NES = 1.35), and one suppressed route, the transforming growth factor-β (TGF-β) signaling pathway (NES = −1.47). The activation of ER-mediated phagocytic routes revealed the upregulation of F-actin and several vATPase transcripts (S1 Fig in S1 File). The induction of NER and NCT pathways is connected to protein synthesis processes, particularly translation, as identified in the GSEA GO analysis. The downregulation of the TGF-β signaling pathway is associated with Smad transcription factors (S2 Fig in S1 File).

### Enrichment analysis after partial knockdown of metalloprotease in tick midgut

The GSEA method for GO BP, MF and CC terms identified 24 significantly enriched gene sets, comprising 8 upregulated and 16 downregulated pathways (S2 Data). Ablation of endogenous metalloprotease led to the upregulation of several biological and functional routes associated with RNA splicing (NES = 1.74, Fig 3B). This process appears to be energetically driven by heightened ATP hydrolysis activity (NES = 1.16) in the mitochondrion (NES = 1.42), catalyzed by nucleases (NES = 1.24) and influenced by iron homeostasis (NES = 1.19). Significantly suppressed pathways were classified into one of the following four categories: detoxification, protein modification, catalytic activity and molecule binding (nucleotide and protein) (Fig 3B). Detoxification processes presented the lowest NES pathway values, with a reduction in peroxidase activity (NES = −1.81) leading to a decreased response to oxidative stress (NES = −1.71) and reduced antioxidant activity (NES = −1.49). In contrast to antigen p23 silencing, catalytic activity following metalloprotease ablation was driven by the suppression of protein kinase activity, responsible for catalyzing phosphorylation. Significant suppression of pathways associated with transcription coregulator activity (NES = −1.30) was also observed. In addition, the GSEA KEGG analysis revealed 3 significantly enriched biochemical pathways (S2 Data), with the activation of phagosome and NER pathways, and the suppression of the Wnt signaling pathway.

### Validation of transcriptomics data

**Fig 4:**
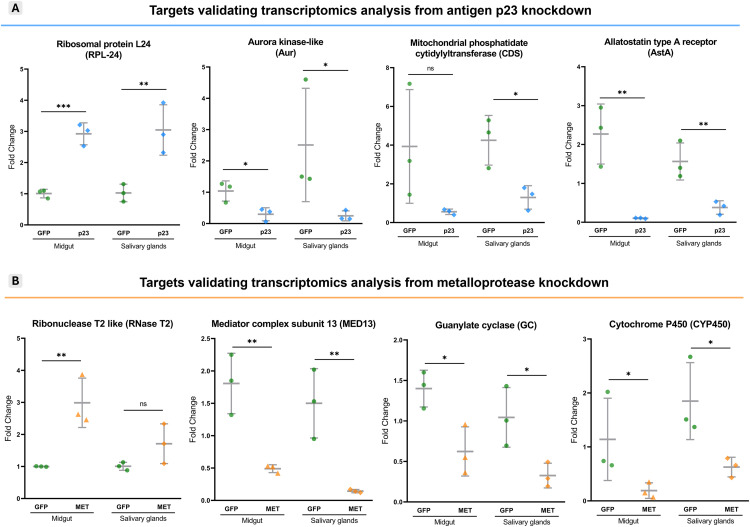
Corroboration of mRNA levels for target differentially expressed genes (DEGs) following antigen p23 (A) and metalloprotease (B) gene knockdown. A total of eight DEGs (four/group) were selected to validate RNA-seq analysis via reverse transcriptase quantitative PCR (RT-qPCR), including two upregulated and six downregulated DEGs, matching the observed expression patterns in midgut. Further analysis of salivary gland tissue detected the same transcriptional profiles in both antigen p23 and metalloprotease-silenced groups. * p < 0.05, ** p < 0.01, *** p < 0.001, ns: not significant.

For the validation of transcriptomics analysis following antigen p23 gene knockdown, four DEGs were selected based on their predicted biological function, comprising one upregulated and three downregulated DEGs (Table 1). While the overexpressed ribosomal protein L24 (RPL-24) was associated with protein formation, the selected suppressed DEGs, aurora kinase-like (Aur), mitochondrial phosphatidate cytidylyltransferase (CDS) and allatostatin type A receptor (AstA) were connected to catalytic activity/protein modification, lipid metabolism and G-protein activity, respectively. RT-qPCR genetic expression matched the results from RNA-seq analysis, revealing a 100% correlation of expression patterns (Fig 4A). Further analysis in the salivary glands indicated that the target gene expression changes observed in this tissue mirrored those seen in the midgut when compared to the GFP control group (Fig 4A).

Transcriptomics data validation following metalloprotease knockdown leaned on the expression of four DEGs, with one upregulated and three downregulated genes (Table 2). While the overexpressed ribonuclease T2 like (RNase T2) was associated with catalytic activity, the selected suppressed DEGs, mediator complex subunit 13 (MED13), guanylate cyclase (GC) and cytochrome P450 (CYP450) were linked to molecule binding, catalytic activity and detoxification. The significant RT-qPCR results also corroborate midgut RNA-seq analysis following the partial knockdown of metalloprotease and match the target expression pattern observed in the salivary glands, where metalloprotease was fully silenced (Fig 4B).

## Discussion

Deepening the understanding of tick physiology and cellular processes can further enhance knowledge of tick-host interactions, which are becoming increasingly important in both medical and veterinary research [44]. This study focused on the functional characterization of salivary and midgut biomolecules antigen p23 (A0A0K8RKR7) and metalloprotease (A0A0K8RCY8) in *I. ricinus* ticks, which are known enablers of the tick feeding process [16,18]. Although silencing the expression of these biomolecules did not result in any significant phenotypic changes in tick feeding and reproduction, midgut transcriptomics analysis revealed several biologically and metabolically relevant changes. Silencing antigen p23 gene expression led to the identification of significantly upregulated pathways associated with protein production, whereas suppressed routes were primarily involved in ion transport, lipid metabolism, catalytic activity, protein modification, and G-protein activity. Partial knockdown of metalloprotease resulted in the upregulation of pathways related to RNA splicing, while significantly suppressed routes associated with detoxification, protein modification, catalytic activity and molecule binding.

Interestingly, silencing the expression of antigen p23 resulted in higher tick detachment rates, which may reflect compensatory upregulation of other salivary components involved in tick feeding or the removal of a regulatory checkpoint associated with it. Alternatively, the absence of host immune or physiological factors due to the use of artificial feeding systems could have affected this result. On the other hand, oviposition was generally low across all experimental groups, with only a small proportion of ticks successfully laying eggs. This finding highlights that silencing the expression of antigen p23 and metalloprotease does not significantly impact tick reproductive fitness. The use of artificial feeding systems may contribute to the overall low oviposition, suggesting that host-derived factors may be critical for egg maturation and deposition. Although antigen p23 and metalloprotease do not appear to be crucial proteins for tick feeding and reproduction, they present allergenic potential in the zebrafish model of AGS and therefore may play a key role in disease pathogenesis [19,20].

Several studies have shown that the recombinant protein antigen p23 from *I. scapularis* exhibits anticoagulant activity, participating in the early stages of blood coagulation by delaying thrombin generation, thus interfering with host coagulation mechanisms to promote optimal tick feeding [18,45,46]. The coagulation cascade further involves a chain of serine proteases that are activated through proteolytic cleavage, resulting in biochemical amplification [47,48]. Our antigen p23 gene knockdown studies in *I. ricinus* revealed the upregulation of six serine-type endopeptidase inhibitors, which act as anti-coagulants [49], thereby providing evidence for functional redundancy. This activity is also supported by the detection of enriched pathways related to heightened protein production and the activation of serine-type peptidases and metallopeptidases, potential modulators of coagulation [47,50]. Moreover, silencing antigen p23 in tick midgut led to the suppression of pathways associated with ion transport, lipid metabolism, catalytic activity, protein modification, and G-protein activity. In this sense, the downregulation of neurotransmitter homeostasis in the midgut could potentially impact nutrient absorption, gut mobility, and the microbiome [51–53]. The inhibition of the PI cellular lipidic molecules could tremendously affect cell regulation by disrupting membrane dynamics, tampering ion transport and altering cellular signaling [54,55]. The suppression of PTP activity could interfere not only with cellular proliferation and differentiation processes [56,57], but also with cellular signaling by catalyzing the dephosphorylation of downstream proteins [58]. Finally, inhibiting the GPCR signaling pathway can potentially alter physiological processes, including stress response and feeding behaviors [59]. The inhibition of this pathway could be correlated with the metabolic activation of ER-mediated phagocytic routes, which potentially targets host cells undergoing necrotic or apoptotic processes [60]. Ultimately, antigen p23 in the tick midgut appears to play a functional role in cell homeostasis, primarily by participating in regulatory and signaling processes essential for cell viability.

Tick metalloproteases exhibit proteolytic, metal-dependent activity and have been shown to regulate blood meal uptake due to their anti-clotting activity at the site of attachment [17,50,61]. *I. ricinus* metalloproteases were found to interfere with blood meal completion *in vivo* and modulate fibrinolysis in salivary glands extracts [16,62]. We detected three functionally redundant, upregulated proteolytic enzymes with carboxypeptidase and endopeptidase activities. The overexpression of these molecules potentially compensates for metalloprotease silencing, and as a result, ticks did not show any changes in feeding patterns, except for slightly lower average weights. Nevertheless, there were significantly suppressed pathways associated with detoxification, protein modification, catalytic activity and molecule binding. The reduction of oxidoreductase activity found in the midgut cells could likely increase their vulnerability to oxidative stress, leading to the buildup of reactive oxygen species (ROS) that could cause cellular damage, impair barrier function, and trigger inflammatory responses [63,64]. Additionally, the downregulation of the Wnt signaling metabolic pathway could be modulated by this oxidative distress context, resulting in reduced cell proliferation and even increased susceptibility to death [65]. On the other hand, the reduction in the catalytic post-translational regulatory process of phosphorylation could impair cellular mechanisms such as protein synthesis, cell division, signal transduction, enzyme activation, and cell growth [66]. Moreover, the suppression of transcription coregulator activities, which are commonly regulated by protein phosphorylation, can further lead to changes in cell behavior [67]. Overall, metalloprotease in the tick midgut appears to be involved in regulating the cellular response to oxidative stress, thereby reducing cell damage and promoting regular cell proliferation, growth and behavior. Nevertheless, these biological and metabolic changes in the tick midgut need to be cautiously interpreted due to the lack of complete metalloprotease gene silencing, possibly due to the larger presence of other protein isoforms in this tissue [16]. However, the target expression patterns analyzed by RT-qPCR in the midgut and the salivary glands fully matched, further strengthening the credibility of our transcriptomics findings and the overall effects observed following RNAi.

## Conclusions and limitations

Although this study advances our understanding of the functional role of tick proteins antigen p23 and metalloprotease, there are several limitations that need to be considered. Viable tick detachment was low and inconsistent in the GFP control group, potentially indicating experimental handling errors that could have influenced the outcomes and affected tick physiology. Moreover, the artificial membrane feeding system does not fully replicate natural tick-host interactions, which may affect the natural regulation of salivary gland proteins and host-pathogen interactions, potentially leading to differences in tick transcriptome compared to those observed under natural feeding conditions on a live host. Mapping the transcriptome directly to *I. ricinus* was not possible, as the required “gff.gz” file for reference-based analyses has not yet been generated for this species. In this sense, *I. persulcatus* was used as reference genome, which may introduce inaccuracies in gene annotation, expression analysis and functional interpretation. Mapping rates were higher with *I. persulcatus* than with *I. scapularis*, supporting its use as a suitable reference genome for this study. However, these limitations were minimized by validating RNA-seq results with RT-qPCR analysis. *De novo* transcriptome assembly was not possible to conduct due to financial constraints. As previously mentioned, the expression of the metalloprotease was not fully silenced in the midgut, therefore the results should be interpreted with caution. Transcriptomics analysis of the salivary gland tissue following RNAi could not be performed, as the RNA samples did not meet the quality control standards required by the sequencing service. However, expression patterns between midgut and salivary glands were similar. Despite these limitations, this study contributes to a broader understanding of tick physiology, particularly the involvement of antigen p23 and metalloprotease in midgut cellular processes. Although assessing transcript reduction provides a direct and reliable measure of RNAi efficacy and has been validated [21,30], future studies should consider using a larger number of ticks to evaluate protein levels in response to RNAi.

Tick-host interactions are shaped by long-term co-evolution and involve diverse tick salivary proteins that modulate the host immune system, hemostasis and tissue repair, key processes in successful tick feeding and pathogen transmission [44]. This study focused on the functional roles of midgut and salivary proteins antigen p23 and metalloprotease in *I. ricinus* females using RNAi. Silencing of antigen p23 did not translate in clear phenotypic changes, possibly due to compensatory mechanisms such as functional redundancy, therefore masking broader physiological effects. Nevertheless, transcriptomic analysis revealed that antigen p23 silencing affected midgut pathways involved in ion transport, lipid metabolism, catalytic activity, protein modification, and G-protein activity, highlighting the role of antigen p23 in maintaining cell homeostasis through regulatory and signaling processes. Interestingly, metalloprotease was fully silenced in the salivary glands but not in the midgut, where the gene expression level resembled that of the GFP control group. No changes were observed in feeding behavior or oviposition, potentially due to functional redundancy or a non-essential role in these functions. Profiling of the midgut transcriptome following metalloprotease silencing revealed suppression of pathways related to detoxification, protein modification, catalytic activity, and molecule binding. This suggests that metalloprotease in the tick midgut participates in the cellular response to oxidative stress, minimizing cell damage and supporting cell survival and proliferation. The study of medically relevant tick proteins and their role in tick physiology paves the way into a binomial course of action. These efforts can either drive the development of new therapeutic agents derived from tick salivary/midgut compounds, or the design of vaccines aimed at blocking pathogen transmission and even AGS sensitization.

## Supporting information

S1 File Supporting Information (S1 Table, S2 Table, S3 Table, S4 Table, S1 Fig., S2 Fig.). (PDF)

S1 Data Transcriptomics data analysis, including DEGs, functional gene profiling, GSEA GO and GSEA KEGG, following antigen p23 gene knockdown by RNAi in female *Ixodes ricinus* tick midgut. (XLSX)

S2 Data Transcriptomics data analysis, including DEGs, functional gene profiling, GSEA GO and GSEA KEGG, following metalloprotease gene knockdown by RNAi in female *Ixodes ricinus* tick midgut. (XLSX)
