# Supplementary material for: Transcriptomics insights into the functional role of tick Ixodes ricinus proteins metalloprotease and antigen p23
Source: PLoS One. 2025 Nov 14;20(11):e0336570. doi: 10.1371/journal.pone.0336570 (PMC12617944; doi:10.1371/journal.pone.0336570)

Supporting information for

Transcriptomics insights into the functional role of tick *Ixodes ricinus* proteins metalloprotease and antigen p23

Rita Vaz-Rodrigues[1], Vincent C. Duru[2,3], Ard M. Nijhof[2,3], José de la Fuente[1,4*]

[1] SaBio (Health and Biotechnology), Instituto de Investigación en Recursos Cinegéticos (IREC, CSIC-UCLM-JCCM), Ciudad Real, Spain

[2] Institute of Parasitology and Tropical Veterinary Medicine, Freie Universität Berlin, Berlin, Germany

[3] Veterinary Center for Resistance Research, Freie Universität Berlin, Berlin, Germany

[4] Department of Veterinary Pathobiology, Center for Veterinary Health Sciences, Oklahoma State University, Stillwater, Oklahoma, United States of America

*Correspondence: José de la Fuente, SaBio, Instituto de Investigación en Recursos Cinegéticos (IREC-CSIC-UCLM-JCCM), Ronda de Toledo 12, 13005 Ciudad Real, Spain. Email: jose_delafuente@yahoo.com

Datasets

S1 Dataset. Transcriptomics data analysis, including DEGs, functional gene profiling, GSEA GO and GSEA KEGG, following antigen p23 gene knockdown by RNAi in female *Ixodes ricinus* tick midgut.

S2 Dataset. Transcriptomics data analysis, including DEGs, functional gene profiling, GSEA GO and GSEA KEGG, following metalloprotease gene knockdown by RNAi in female *Ixodes ricinus* tick midgut.

Tables

S1 Table. Primer sequences used for validation of target differentially expressed genes (DEGs) mRNA expression levels following antigen p23 gene knockdown in *Ixodes ricinus* tick midgut.

Target	IDs	Amplicon size	Primer forward (5’-3’)	Primer reverse (5’-3’)
Ribosomal protein L24 (RPL-24)	HPB47_017850 IPEI_008270	131	ACTACTGGACGATTCACGGACTG	GAGGGAGGGCCAGTACTTGTT
Aurora kinase-like (Aur)	HPB47_014629 IPEI_004155	186	CAGATTGGCCAGATCATGGTGATG	CAGCTCTCCTTTGGGTGCGTA
Allatostatin type A receptor (AstA)	HPB47_005012 IPEI_017506	112	AACAGAGGGCGGAAGTTTTGAA	TATGGATTCTGGCAGAGCCGTC
Mitocondrial phosphatidate cytidylyltransferase (CDS)	HPB47_023844 IPEI_010956	77	CGGCGTTGTCTGGAGATCAAGTT	AGTGAAGGGAATTCCACAGACCTGC

S2 Table. Primer sequences used for validation of target differentially expressed genes (DEGs) mRNA expression levels following metalloprotease partial gene knockdown in *Ixodes ricinus* tick midgut.

Target	IDs	Amplicon size	Primer forward (5’-3’)	Primer reverse (5’-3’)
Ribonuclease T2 like (RNase T2)	HPB47_001654 IPEI_008270	131	ACTACTGGACGATTCACGGACTG	GAGGGAGGGCCAGTACTTGTT
Mediator complex subunit 13 (MED13)	HPB47_027399 IPEI_017802	159	GCCTCTTCCACGACCGCTAT	TGTGGTCCTGCTGGAAGGTG
Guanylate cyclase (GC)	HPB47_008153 IPEI_016458	140	TACTGCTCAGGGCAAGGAAGACA	CCTGGAAAGTTCGATCGTCGTGC
Cytochrome P450 (CYP450)	HPB47_019282 IPEI_017052	119	CATGATCGAAGCCAAGCAGTCC	TAAACCCGTTGGCGAGCTGTGA

S3 Table. Main Gene Ontology (GO) Biological Processes (BP) and Molecular Function (MF) pathways associated with the upregulated (up) and downregulated (down) genes from antigen p23 gene knockdown in *Ixodes ricinus* tick midgut.

GO term	GO type	Number of genes	Gene expression	Gene IDs	Observations
proteolysis	BP	7	up	HPB47_009280, HPB47_004434,HPB47_028387,HPB47_005579, HPB47_028388, HPB47_024456, HPB47_014190	Associated with serine-type peptidase activity (N=3, MF) and metallopeptidase activity (N=2, MF).
carbohydrate metabolic process	BP	5	up	HPB47_020144, HPB47_017917, HPB47_013911, HPB47_003950, HPB47_021555	Associated with hydrolase activity (N=3, MF).
regulation of transcription	BP	4	up	HPB47_000632, HPB47_006896, HPB47_014243, HPB47_027004	
immune response	BP	3	up	HPB47_012122, HPB47_001261, HPB47_023216	
toll-like receptor signaling pathway	BP	2	up	HPB47_001261, HPB47_023216	Associated with transmembrane signaling receptor activity (N=2, MF) and membrane (N=2, CC).
translation	BP	2	up	HPB47_017850, HPB47_006933	Associated with the ribosome (N=2, CC).
protein binding	MF	6	up	HPB47_021759, HPB47_021377, HPB47_014374, HPB47_024039, HPB47_013683, HPB47_021685	
GTPase activity & GTP binding	MF	3	up	HPB47_016592, HPB47_013858, HPB47_020564	
RNA binding	MF	5	up	HPB47_022399, HPB47_016263, HPB47_010051, HPB47_009808,HPB47_017850	
serine-type endopeptidase activity	MF	6	up	HPB47_004434,HPB47_007311,HPB47_003913,HPB47_005579,HPB47_027208,HPB47_014190	Associated with inhibitor activity (N=3, BP), proteolysis (N=3,BP) and extracellular region/space (N=6, CC).
zinc ion binding	MF	5	up	HPB47_000031,HPB47_016562,HPB47_011242,HPB47_012122,HPB47_006738	
transmembrane transport	BP	13	down	HPB47_017384, HPB47_028149, HPB47_002496, HPB47_012141, HPB47_016039, HPB47_024310, HPB47_013081, HPB47_007177, HPB47_024544, HPB47_023796, HPB47_020148, HPB47_024954, HPB47_007139	Associated with transmembrane transporter activity (N=10, MF) and ABC-type transporter activity (N=2, MF).

G protein-coupled receptor signaling pathway	BP	9	down	HPB47_003370, HPB47_006483, HPB47_024347, HPB47_000165, HPB47_001957, HPB47_000106, HPB47_005012, HPB47_023493, HPB47_008007	Associated with G protein-coupled receptor activity (N=9, MF) and membrane (N=9, CC).
protein phosphorylation	BP	8	down	HPB47_010640, HPB47_011909, HPB47_012298, HPB47_000550, HPB47_021990, HPB47_020216, HPB47_014629, HPB47_009968	Associated with protein kinase activity (N=8, MF) and ATP binding (N=8, MF).
proteolysis	BP	8	down	HPB47_001891, HPB47_009470, HPB47_002123, HPB47_022778, HPB47_022347, HPB47_007825, HPB47_001820, HPB47_001931	Associated with serine (N=3), cysteine-type (N=1) peptidases and metalloendopeptidases (N=4), MF.
regulation of transcription, DNA-templated	BP	7	down	HPB47_015713, HPB47_012603, HPB47_022967, HPB47_009375, HPB47_003496, HPB47_011459, HPB47_005238	Associaded with DNA-binding (N=4, MF).
ion transmembrane transport	BP	5	down	HPB47_024787, HPB47_005065, HPB47_010398, HPB47_023274, HPB47_013925	
signal transduction	BP	3	down	HPB47_004501, HPB47_014627, HPB47_005973	
protein binding	MF	34	down	HPB47_021805, HPB47_000324, HPB47_003404, HPB47_027791, HPB47_004501, HPB47_027536, HPB47_019300, HPB47_006380, HPB47_008404, HPB47_003496, HPB47_012563, HPB47_013116, HPB47_004556, HPB47_009389, HPB47_002353, HPB47_025725, HPB47_024325, HPB47_023221, HPB47_026916, HPB47_010181, HPB47_013759, HPB47_001698, HPB47_003028, HPB47_008694, HPB47_025024, HPB47_003940, HPB47_019693, HPB47_020744, HPB47_027677, HPB47_027383, HPB47_024656, HPB47_004727, HPB47_008723, HPB47_000005	
ATP binding	MF	14	down	HPB47_023796, HPB47_018040, HPB47_016039, HPB47_010377, HPB47_007975, HPB47_021662, HPB47_024649, HPB47_002020, HPB47_021875,	

				HPB47_017632, HPB47_000550, HPB47_010640, HPB47_014629, HPB47_009968	
DNA binding	MF	8	down	HPB47_002020, HPB47_018169, HPB47_027567, HPB47_015720, HPB47_012603, HPB47_011459, HPB47_005238, HPB47_009375	
calcium ion binding	MF	5	down	HPB47_026107, HPB47_025776, HPB47_007882, HPB47_011642, HPB47_000170	Associated with extracellular region (N=3, CC).
chitin binding	MF	4	down	HPB47_020474, HPB47_015297, HPB47_000445, HPB47_020182	

S4 Table. Main Gene Ontology (GO) Biological Processes (BP) and Molecular Function (MF) pathways associated with the upregulated (up) and downregulated (down) genes from metalloprotease gene knockdown in *Ixodes ricinus* tick midgut.

**GO term**	**GO type**	**Number of genes**	**Gene expression**	**Gene IDs**	**Observations**
G protein-coupled receptor signaling pathway	BP	2	up	HPB47_008629, HPB47_012110	Associated with G protein-coupled receptor activity (N=2, MF) and membrane (N=2, CC).
lipid metabolic process	BP	2	up	HPB47_016435, HPB47_009100	Associated with sphingomyelin phosphodiesterase activity (N=1, MF) and lipase activity (N=1, MF).
microtubule cytoskeleton organization	BP	2	up	HPB47_024157, HPB47_016586	
proteolysis	BP	3	up	HPB47_026288, HPB47_010253, HPB47_028387	
transmembrane transport	BP	8	up	HPB47_015706, HPB47_026124, HPB47_012269, HPB47_021038, HPB47_021043, HPB47_020148, HPB47_025631, HPB47_012893	Associated with transmembrane transporter activity (n=6, MF) and membrane (n=5, CC).

acireductone dioxygenase [iron(II)-requiring] activity	MF	2	up	HPB47_013115, HPB47_005701	
catalytic activity	MF	2	up	HPB47_022333, HPB47_010928	
chitin synthase activity and hexosyltransferase activity	MF	2	up	HPB47_011812, HPB47_011813	
nucleic acid binding	MF	4	up	HPB47_004428, HPB47_010924, HPB47_022760,HPB47_012290	
oxidoreductase activity	MF	5	up	HPB47_028342, HPB47_019589, HPB47_024631, HPB47_012290, HPB47_000961	Associated with iron ion binding (N=3, MF) and heme binding (N=2, MF).
protein binding	MF	3	up	HPB47_019737, HPB47_027967, HPB47_014374	
RNA binding	MF	3	up	HPB47_001654, HPB47_010185, HPB47_010184	
sulfotransferase activity	MF	6	up	HPB47_008140, HPB47_007947, HPB47_023646, HPB47_004800, HPB47_027299, HPB47_027456	
dolichol-linked oligosaccharide biosynthetic process	BP	2	down	HPB47_010658, HPB47_006305	
ion transmembrane transport	BP	3	down	HPB47_024787, HPB47_023274, HPB47_019985	Associated with transmembrane signaling receptor activity (N=3, MF) and ligand-gated ion channel activity (N=3, MF)
proteolysis	BP	4	down	HPB47_026112, HPB47_022268, HPB47_022347, HPB47_005600	Associated with serine (N=1), cysteine-type (N=1) peptidases and metalloendopeptidases (N=2), MF.
calcium ion binding	MF	2	down	HPB47_021962, HPB47_010653	
oxidoreductase activity	MF	3	down	HPB47_020587, HPB47_019282, HPB47_021618	
protein binding	MF	7	down	HPB47_026916, HPB47_022790, HPB47_024591, HPB47_019041, HPB47_020098, HPB47_023597, HPB47_022347	

Figures

S1 Fig. Activation of ER-mediated phagocytic pathway from the Kyoto Encyclopedia of Genes and Genomes (KEGG) database highlighting the upregulation of F-actin and multiple vATPase transcripts following antigen p23 gene knockdown via RNA interference. This pathway-based data integration and visualization was performed using the *Pathview* package in R (version 4.3.2).

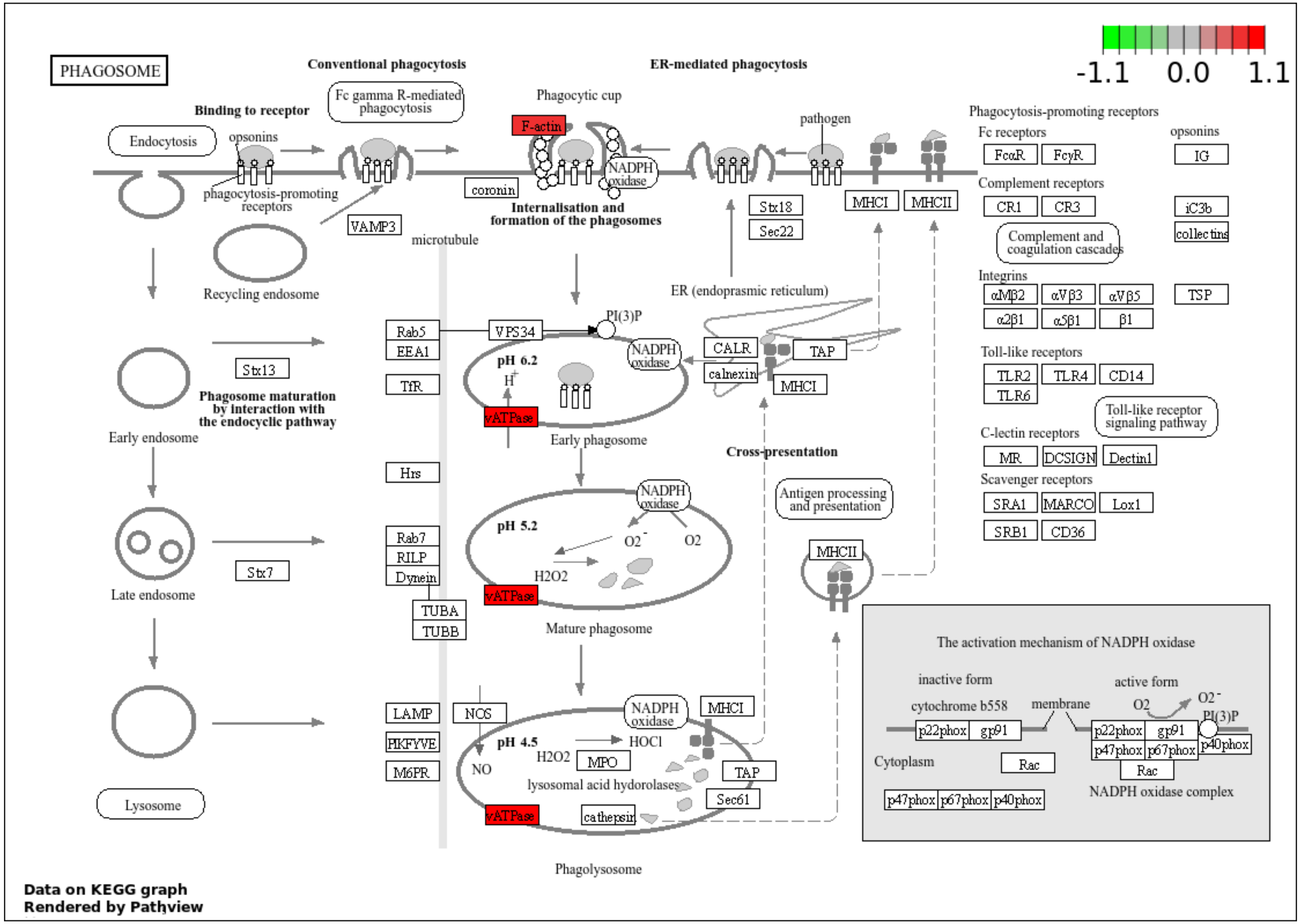

S2 Fig. Suppression of the transforming growth factor-β (TGF-β) signaling pathway from the Kyoto Encyclopedia of Genes and Genomes (KEGG) database highlighting the downregulation of Smad transcription factors following antigen p23 gene knockdown via RNA interference. This pathway-based data integration and visualization was performed using the *Pathview* package in R (version 4.3.2).

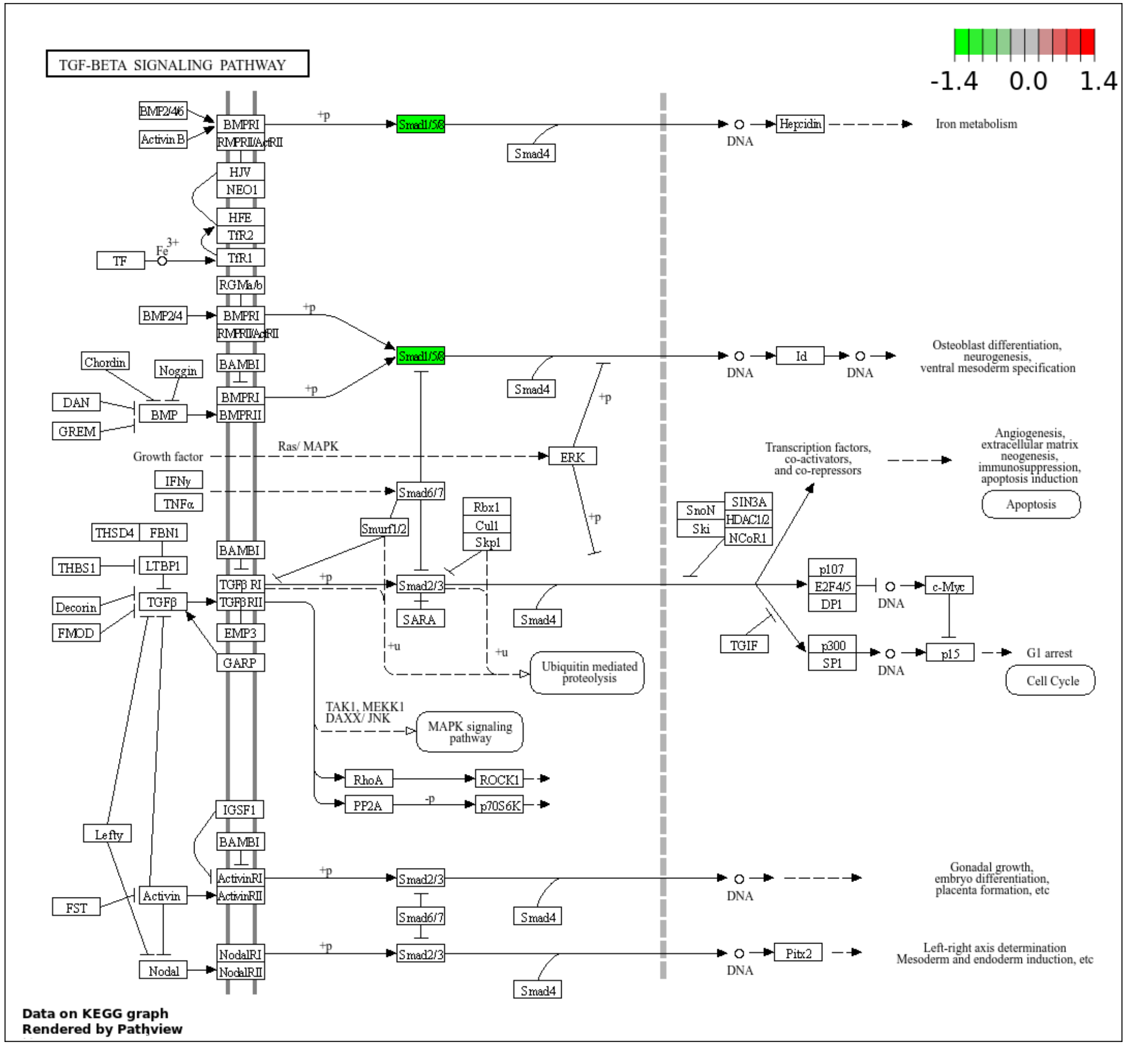

Supplement: S1 File — (PDF) [file pone.0336570.s001.pdf]
